# Cytotoxicity and variant cellular internalization behavior of water-soluble sulfonated nanographene sheets in liver cancer cells

**DOI:** 10.1186/1556-276X-8-208

**Published:** 2013-05-02

**Authors:** Stuart J Corr, Mustafa Raoof, Brandon T Cisneros, Oleksandr Kuznetsov, Katheryn Massey, Warna D Kaluarachchi, Matthew A Cheney, Edward W Billups, Lon J Wilson, Steven A Curley

**Affiliations:** 1 Department of Surgical Oncology, University of Texas M. D. Anderson Cancer Center, Unit 107, Rm. T4.3936, 6767 Bertner, Houston, TX, 77030, USA; 2 Department of Chemistry, Rice University, Houston, TX, 77005, USA; 3 Richard E. Smalley Institute for Nanoscale Science and Technology, Rice University, Houston, TX, 77005, USA; 4 Department of Physics and Astronomy, Rice University, Houston, TX, 77005, USA; 5 Department of Mechanical Engineering and Materials Science, Rice University, Houston, TX, 77005, USA; 6 Division of Surgery, University of Texas M. D. Anderson Cancer Center, Unit 1447, 1400 Pressler Street, Houston, TX, 77230-1402, USA

**Keywords:** Cytotoxicity, Sulfonated graphene sheets, Cancer cells

## Abstract

Highly exfoliated sulfonated graphene sheets (SGSs), an alternative to graphene oxide and graphene derivatives, were synthesized, characterized, and applied to liver cancer cells *in vitro*. Cytotoxicity profiles were obtained using 3-(4,5-dimethylthiazol-2-yl)-2,5-diphenyltetrazolium bromide, WST-1[2-(4-iodophenyl)-3-(4-nitrophenyl)-5-(2,4-disulfophenyl)-2*H*-tetrazolium, and lactate dehydrogenase release colorimetric assays. These particles were found to be non-toxic across the concentration range of 0.1 to 10 μg/ml. Internalization of SGSs was also studied by means of optical and electron microscopy. Although not conclusive, high-resolution transmission and scanning electron microscopy revealed variant internalization behaviors where some of the SGS became folded and compartmentalized into tight bundles within cellular organelles. The ability for liver cancer cells to internalize, fold, and compartmentalize graphene structures is a phenomenon not previously documented for graphene cell biology and should be further investigated.

## Background

Carbon-derived nanoparticles (NPs) such as single- and multi-walled carbon nanotubes, fullerenes, and graphene are all receiving attention because of their interesting and unusual electronic [1], thermal [2], and mechanical [3] properties. We have recently demonstrated a facile route towards the synthesis of nanosized water-soluble sulfonated graphene sheets (SGSs) that use graphite as the starting material [4]. This method relies on the addition of phenyl radicals with subsequent sulfonation of the phenyl groups and produces fewer defects and holes that can be introduced into the graphene plates through the use of heavy sonication. A possible application of these SGSs is within the medical sector due to their enhanced solubility (compared to other graphene derivatives) and potential for surface modifications for attachment of biomolecules and drugs. However, the interaction of SGSs with biological systems has yet to be investigated and is the basis of the work described herein.

To date, much of the biological work regarding graphene has focused on assessing the cytotoxicity, cell adhesion, proliferation, and antibacterial properties of *graphene oxide* (GO) [5-8] as well as biodistribution, toxicology, and internalization of various suspensions of GO complexes. These include ^125^I and ^188^Re radioisotope-labeled GO [9,10], PEGylated GO for cellular imaging and delivery of water-insoluble cancer drugs [11-13], and the imaging and treatment of brain, lung, and breast xenograft tumors in mice through the use of photothermal light therapy from the absorption of near-infrared (NIR) light by PEGylated GO with fluorescent Cy7 probes [14].

Toxicity analysis (*in vitro*) of GO (prepared using chemical vapor deposition or the modified Hummers method [15]) on lung [16,17] and neuronal [18] cell lines (A549 and PC12, respectively) has shown concentration-dependent cytotoxicity. The exact mechanism of cell death from GO remains uncertain although a slight increase in lactate dehydrogenase (LDH) from cells, generation of reactive oxygen species, and weak activation of a caspase-3-mediated apoptosis pathway have all been reported. These reports suggest GO cytotoxicity from either direct cellular membrane damage or activation of natural cellular suicide mechanisms.

Similarly, *in vivo* mouse toxicology studies have shown that GO nanoplatelets of diameters 10 to 700 nm apparently cause no acute toxicities at low doses [9,10]. However, at high doses (10 mg/kg), significant pathological changes such as granulomatous lesions, pulmonary edema, inflammatory cell infiltration, and fibrosis were observed throughout the lungs.

In light of the potential applications of graphene materials in drug delivery, imaging, and thermal therapy, but with limitations due to cytotoxicity of GO, we sought to investigate the *in vitro* interaction of our highly water-soluble SGS with liver cancer cells. Our initial studies using the standard 3-(4,5-dimethylthiazol-2-yl)-2,5-diphenyltetrazolium bromide (MTT), WST-1[2-(4-iodophenyl)-3-(4-nitrophenyl)-5-(2,4-disulfophenyl)-2*H*-tetrazolium (WST-1), and LDH colorimetric assays have shown that SGSs are non-toxic up to concentrations of 10 μg/ml. We also show that liver cancer cell lines (SNU449 and Hep3B) can internalize SGSs of diameters up to 5 μm, which in some cases are comparable to the size of the cells themselves. Preliminary electron microscopy analysis also suggests that these cells are capable of folding and compartmentalizing sheets of smaller sizes (approximately 1.41 μm) although more work should be undertaken to validate.

Since graphene has been documented to be the hardest material known [3], this unique behavior of water-soluble SGS with cells is counterintuitive and suggests a novel finding that may have far-reaching applications in biology and medicine such as enhanced drug delivery (due to the large graphene surface area), and should warrant further investigation. Given that these SGSs are non-toxic up to 10 μg/ml, we feel they can be used as an adequate scaffold to simultaneously attach targeting moieties such as EGFR antibodies (e.g., cetuximab, C225) and chemo-agents such as doxorubicin and gemcitabine in a bid to treat hepatocellular carcinoma legions. The use of a targeted thermal ‘trigger’ such as photon activation (i.e., NIR light) or radiofrequency electric fields could allow these SGSs to release their cargo into the cells upon irradiation by a stimuli. Such a scheme has recently been reported using cisplatin-filled ultra-short carbon nanotubes that release their cargo upon exposure to high-intensity radiofrequency electric fields [19].

## Methods

### Sample preparation and characterization

Samples were obtained from Mukherjee et al. [4]. In their technique, highly exfoliated SGSs can be synthesized by sulfonation of commercially available graphite (particle size < 20 μm) in oleum to overcome the cohesive van deer Waals attractions between adjacent sheets. Their exfoliation method was selected over the procedure by Si et al. [20] as it produces fewer defects and holes that can be introduced into the graphene plates through the use of heavy sonication. In brief, the addition of benzoyl peroxide to a suspension of graphite in benzene at 75°C to 80°C provided phenylated graphite, the sulfonation of which by oleum leads to highly-exfoliated graphene sheets which can be further converted into a sodium salt by the addition of 1 M sodium hydroxide. This material, in powder form, is highly soluble in water (approximately 2.1 mg/ml) due to the p-sulphonated substituents, and it is relatively free of basal plane defects that typically result from the removal of the oxygen functionality of comparable GO compounds.

The SGSs in powder form were characterized via Raman spectroscopy, thermogravimetric analysis (TGA), X-ray photoelectron spectroscopy (XPS), and atomic force microscopy (AFM). Raman spectra of the initial graphite material were compared to SGSs using a Renishaw 1000 micro-Raman system (Gloucestershire, UK) with a 514-nm excitation laser source. Multiple spectra were taken [3-5] and normalized to the G band. TGA data were taken using a model SDT 2960 TA (TA Instruments, Newcastle, DE, USA) instrument in both an argon and air atmosphere. Samples were first degassed at 80°C and then heated at 10°C/min to 700°C and held there for 20 min. This allowed for accurate percentage determination of the sodium sulfonate groups (approximately 6%). XPS data were obtained using a physical electronics (PHI QUENTERA, Chanhassen, MN, USA) XPS/ESCA system with a base pressure of 5 × 10^−9^ Torr. A monochromatic Al X-ray source at 100 W was used with a pass energy of 26 eV and a 45° takeoff angle. The beam diameter was 100.0 μm. Low- and high-resolution survey scans of the elements C, O, Na, and S were taken. At least two separate locations were analyzed for each sample. For AFM studies, aqueous solution of SGSs at 50 mg/l was drop-cast onto freshly cleaved mica and placed in a desiccator for 24 h prior to imaging. Tapping-mode AFM images were taken in air under ambient conditions on a Digital Instruments Nanoscope IIIA (Digital Instruments, Tonawanda, NY, USA).

### Cell culture studies

SGS cytotoxicity was investigated using multiple assays. Cell membrane integrity was evaluated using a LDH release assay. Cell proliferation/metabolic activity was investigated using the popular MTT and WST-1 colorimetric assays. For *in vitro* experiments, approximately 3 mg of the SGS powder was added to 3 ml of phosphate-buffered saline (PBS) to create two suspensions of concentration 1,000 μg/ml. All samples were sterilized for 20 min using a bench-top UV sterilizer. SNU449 and Hep3B liver cancer cells were utilized for the experiments (American Type Culture Collection, Bethesda, MD, USA). The cells were maintained in standard culture conditions with 10% fetal calf serum and penicillin/streptomycin at 37°C. Cell morphology was analyzed using real-time bright-field optical imaging.

#### MTT assay

SNU449 and Hep3B cells were plated in 96-well plates at a density between 1,000 to 2,000 cells per well. After 24 h, the SNU449 and Hep3B cells were exposed to increasing concentrations (0.1, 1.0, 10, and 100 μg/ml) of SGSs in PBS and were compared to a PBS only control group (all suspensions were lightly sonicated for 5 min before use). Cell viability was assessed at 24, 72, and 120 h after exposure to the SGSs. At each time point, the media (100 μl) was carefully aspirated and replaced before adding MTT reagent to each well and incubating for 4 h. The media was again carefully removed, and purple formazan crystals were dissolved in dimethyl sulfoxide (DMSO). The 96-well plates were then spun down at 3,500 rpm for 5 min (to force any cells/SGS debris to the bottom of the well) where 50 μl of the colored media was withdrawn and placed into a fresh 96-well plate. Absorbance was interpreted at 570 nm for each well using a SPECTROstar Nano plate reader (BMG Labtech Inc., Cary, NC, USA).

#### WST-1 assay

These studies were prepared similar to the MTT assay but for a shorter duration (24, 48, and 72 h) as MTT assays showed that maximum toxicity occurred at 72 h. Also, it was harder to keep the control cells from overgrowing for times greater than 72 h. At each time point, WST-1 reagent was added to each well and incubated for 3 h. The 96-well plate was then spun down at 3,500 rpm for 5 min (to force any cells/SGS debris to the bottom of the well) where 50 μl of the colored media was withdrawn and placed into a fresh 96-well plate. This negated any effects from inherent SGS absorption as all the SGSs were contained at the bottom of the discarded well. Absorbance was interpreted at 450 nm for each well using a SPECTROstar Nano plate reader (BMG Labtech Inc.).

#### LDH assay

SNU449 and HEP3B cells were exposed to various concentrations of SGSs (0.1, 1.0, 10.0, and 100 μg/ml) for 24, 48, and 72 h, and the cell-free supernatant was removed. Maximum LDH release was obtained by exposing the cells to a 2% Triton-X 100 solution to permeabilize the membranes. LDH activity was determined by the use of a cytotoxicity detection kit purchased from Roche Applied Science (Indianapolis, IN, USA). Aliquots of the cell culture media from the SGS-exposed samples, untreated samples, and the permeabilized samples were added to a 96-well plate, and an equal volume of LDH cytotoxicity detection reagent was added. The 96-well plates were read on a spectrophotometer, and the absorbance at 492 nm was measured. Calculations were performed as per the recommendations of the kit. To show that SGS does not interfere with the kit, cells were permeabilized with a 2% Triton-X 100 solution. The lysate was incubated with various concentrations of SGS for 24 h. No difference was observed for any of the control samples indicating that SGSs do not interfere with the assay.

#### Flow cytometry

Viability was measured with flow cytometry (LSRII, BD Biosciences, Franklin, NJ, USA) as described previously [21]. Briefly, cell media was aspirated, and the adherent cells were collected after trypsinization. Each sample was washed and stained with annexin V-FITC and propidium iodide (PI) without fixation or permeabilization. Annexin V is a protein that binds to phosphatidylserine, which is externalized in apoptotic cells. Propidium iodide fluoresces when it is bound to DNA in membrane-damaged cells. Cells that were negative for both markers were characterized as viable. Approximately 50,000 events were measured for each sample. Due to sample availability, only one time point (24 h) was measured on one cell line (SNU449) at two concentrations (10 and 100 μg/ml). As such, these data have been placed in the Additional file 1.

#### Real-time optical bright-field microscopy

Hep3B cells were cultured in glass bottom (no. 1.5) 24-well plates purchased from MatTek Corporation (Ashland, MA, USA). After overnight incubation, the cells formed non-confluent monolayers. The 24-well plate was placed in an incubator enclosing a 1X81 Olympus microscope (Center Valley, PA, USA) equipped with a DSU Confocal Attachment and a ×60 oil immersion objective. The cells were allowed to equilibrate with the incubator environment (37°C, 5% CO_2_) before adding pre-warmed SGSs and acquiring images. Eight *Z*-plane images were acquired with a gap of 1 μm every 15 min. A typical experiment comprised of 10 to 15 waypoints. In-focus light from all planes was merged and is represented in the still shots and the movies. Hep3B cells with no exposure to SGS were also imaged as a control.

### Transmission/scanning electron microscopy

For transmission electron microscopy (TEM) imaging, 25,000 Hep3B or SNU449 cells were plated in 12-well plates. After 24 h, the cells were exposed to the SGS at 10 μg/ml for 24 h. The media was removed, and cells were washed twice with PBS. The cells were then harvested after trypsinization and washed once more with PBS. Finally, the cells were resuspended in Trump’s Fixative (BBC Biochemical, Seattle, WA, USA). Samples were washed with 0.1% cacodylate-buffered tannic acid, treated with 1% buffered osmium tetroxide, and stained with 1% uranyl acetate. The samples were ethanol dehydrated and embedded in LX-112 medium. After polymerization, the samples were cut with an UltraCut E Microtome (Leica, IL, USA), double stained with uranyl acetate/lead citrate in a Leica EM stainer, and imaged with a JEM 1010 TEM (Jeol USA, Inc., Peabody, MA, USA) at an accelerating voltage of 80 kV. Images were acquired with an AMT Imaging System (Advanced Microscopy Techniques Corp., Woburn, MA, USA). For SEM, the cells were prepared in a similar manner. The dried samples were coated with a 35-nm-thick platinum layer. Samples were imaged using a JSM 5900 scanning electron microscope (JEOL USA, Inc.) equipped with a backscatter electron detector and digital camera. The beam energy was 5 kV.

## Results and discussion

### SGS characterization

**Figure 1:**
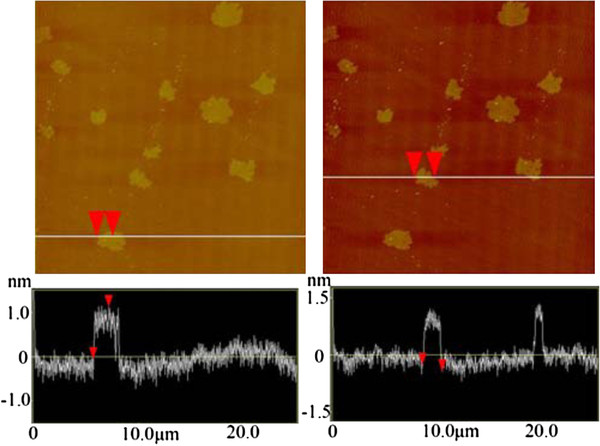
**AFM images of the SGSs.** Left and right images depict completely exfoliated SGSs of diameter 1.41 ± 0.08 μm and height 1.01 ± 0.02 nm. Larger, more graphitic-like materials of diameters approximately 3 to 5 μm were also present in lower quantities (Additional file 1: Figure S1).

As can be seen in Figure 1, AFM statistical analysis showed the majority of SGSs (sample size 61) to be approximately 1.41 ± 0.08 μm in diameter with a height of approximately 1.01 ± 0.02 nm, indicating mainly individualized SGSs [22,23]. In some instances, there was also evidence of larger SGSs of diameter approximately 5.5 μm (Additional file 1: Figure S1). Raman spectra of the initial graphite material and an SGS sample are depicted in Additional file 1: Figure S2. According to previous Raman studies [4], graphene can be identified by monitoring the position of the 2D band, whereby sulfonation of the phenyl groups and subsequent formation of the SGS sodium salt lead to repulsive interactions between the SO_3_− groups (to produce exfoliation), as evidenced by a slight shift in the 2D peak in Additional file 1: Figure S2. Functionalization by sulfonation has also been confirmed by XPS and TGA, which is provided in Additional file 1: Figures S3 and S4, respectively. Taken together, these data characterize the SGS samples as being made up of both individualized SGSs and stacked SGSs of diameters ranging from 1.41 to 5.5 μm.

### Cytotoxicity profiles of SGSs

**Figure 2:**
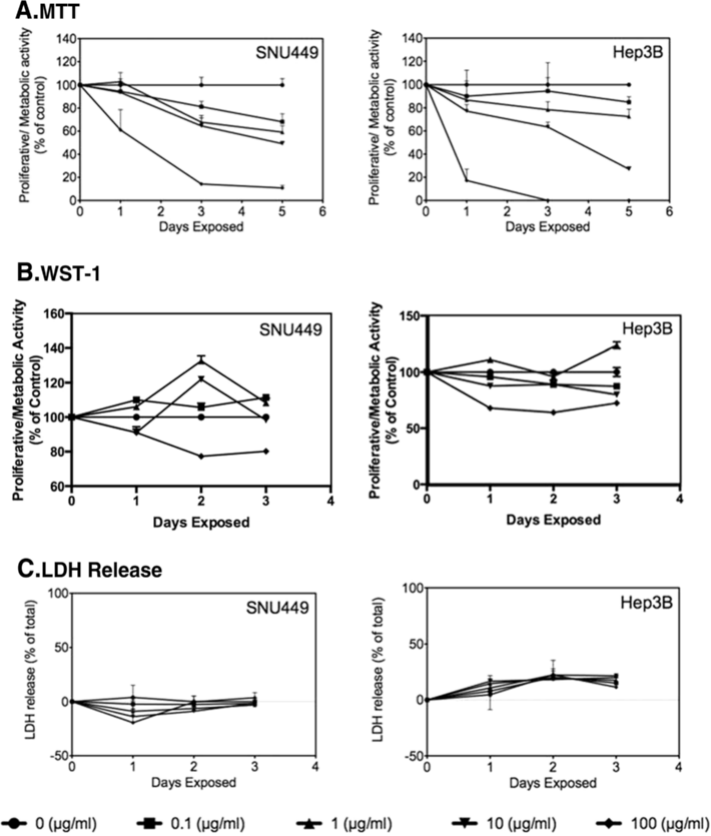
**Cytotoxicity Data (MTT, WST-1, and LDH).** MTT (**A**), WST-1 (**B**), and LDH (**C**) assays of SNU449 and Hep3B cancer cell lines. As a function of time and SGS concentration.

MTT assay analysis over 5 days showed both a time- and concentration-dependent cytotoxicity profile for both cell lines. Control samples were also used in conjunction with the *in vitro* samples to take into account an increase in 570-nm photon absorption due to the SGSs themselves, which could obscure correct interpretation of the results. As can be seen in Figure 2A, although the SNU449 and Hep3B cell lines were approximately 80% to 90% viable after 24 h upon exposure to SGS concentrations of 0.1 to 10 μg/ml, the highest concentration of 100 μg/ml resulted in a drastic drop in viability to 60% and 20% for SNU449 and Hep3B cells, respectively. This decrease in viability occurred over time until almost complete necrosis of cells at 72 h. For lower concentrations, while the Hep3B cells seem to tolerate SGS better, the SNU449 cells had the greater viability (approximately 50%) for the 10 μg/ml concentration after a 5-day period. The WST-1 results shown in Figure 2B depict both a weak concentration- and time-dependent cytotoxicity profile. The viability of Hep3B cells generally stays within the 90% range and only decreases to approximately 70% for the highest concentration. This is also similar for the SNU449 cells which show a constant viability of approximately 90% to 135% for concentrations 0.1 to 10 μg/ml and a loss in viability down to 80% after a period of 48 to 72 h for the maximum concentration of 100 μg/ml. Finally, the release of intracellular LDH can provide evidence of plasma membrane damage. Figure 2C shows minimal membrane damage as evidenced by minimal LDH release in both cell lines after 72 h of exposure to SGS for concentrations up to 100 μg/ml.

Previous work by Zhang et al. [18] demonstrated a similar MTT concentration-dependent viability profile with neural phaeochromocytoma-derived PC12 cells exposed to graphene synthesized via CVD (purified using a diluted hydrochloric acid wash with sonication). They showed cell viability of approximately 40% after 24 h of exposure to their graphene particles at a concentration of 100 μg/ml, which is similar to MTT values seen in this work. In comparison, Chang et al. also demonstrated a concentration-dependent profile which was however not time dependent since they observed similar viability profiles at 24, 48, and 72 h [16].

Although the MTT and WST-1 profiles are generally identical for time periods 24 to 72 h (with possibly the exception of the WST-1 results which show a weak time-dependent and concentration-dependent response), the major difference is the drastic loss in viability for concentrations of 100 μg/ml observed in the MTT assay. This observation could be explained by interactions of SGSs with insoluble MTT formazan crystals (formed after the enzymatic reduction of MTT within the cells) which stabilize their structure and prevent them from becoming solubilized by DMSO. This has already been observed by Wörle-Knirsch et al. [24]. In their work, they showed that single-walled carbon nanotubes (SWNTs) were found to be non-toxic when using assays such as LDH, annexin V, and PI staining, mitochondrial membrane potential, as well as other tetrazolium salt-based water-soluble assays such as WST-1, XTT, or INT. However, the MTT assay was the only assay which displayed SWNT cytotoxicity.

**Figure 3:**
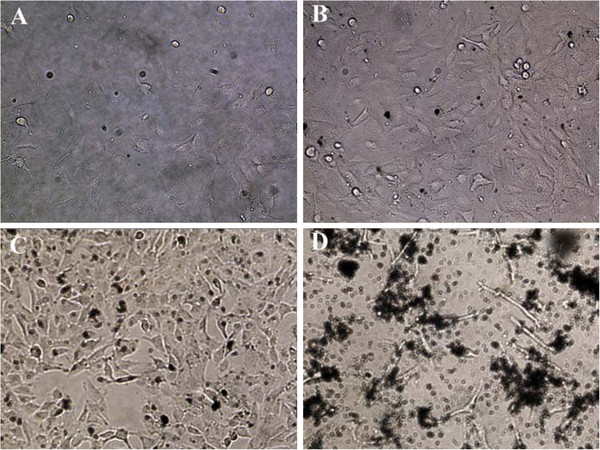
**Optical images of SNU449 cells exposed to SGSs for 72 h.** Images depict control cells (no SGSs) (**A**) and 1 (**B**), 10 (**C**), and 100 (**D**) μg/ml concentrations.

In addition, real-time bright-field microscopy (Figure 3) did not show any morphological features suggestive of cytotoxicity, such as blebbing, membrane rupture, pyknosis, or fragmentation, for concentrations 1 to 10 μg/ml. Also, several cells were observed undergoing mitosis (data not shown). These findings suggest that at these low concentrations, the sulfonation process affords protection to cells against the cytotoxic effects of graphene, similar to the observed protein corona-mediated mitigation of GO cytotoxicity recently published by Hu et al. [17]. However, there was a drastic change in cell morphology for concentrations of 100 μg/ml which shows evidence of pyknosis and fragmented, spindle-like cell features for the SNU449 cell lines. In these regard, we suggest that 10 μg/ml should be the upper concentration limit when using SGSs for full biocompatibility and that more work should be undertaken to understand the exact death mechanism of SGSs at concentrations >10 μg/ml. We initially sought to investigate this through the use of propidium iodide and annexin V FITC staining with cell flow cytometry, but as mentioned in the ‘Methods’ section, we could only perform one time slot (24 h) with one cell line (SNU449) at two concentrations (10 and 100 μg/ml).

Propidium iodide is a cell impermeable fluorophore that can bind to the DNA of cells which have lost nuclear and plasma membrane integrity. From our fluorescence-activated cell sorting (FACS) analysis shown in Additional file 1: Figure S5, we found that with an increasing concentration of SGS nanoparticles, the intensity of positive PI-stained cells increased from approximately 1.9% to 10.3%, suggesting slight cell membrane structural damage, while the majority of cells remain healthy and viable at approximately 93% ± 2.4%. Phosphatidylserine (PS) externalization is an early event in the apoptosis cascade. Annexin V binds to PS with high affinity. Our FACS analysis hence also demonstrates that very few cells were annexin V positive 24 h after exposure to SGS which ruled out apoptosis as a significant cell death mechanism, as was similarly reported for GO materials [16,18].

### Cellular internalization of SGSs

**Figure 4:**
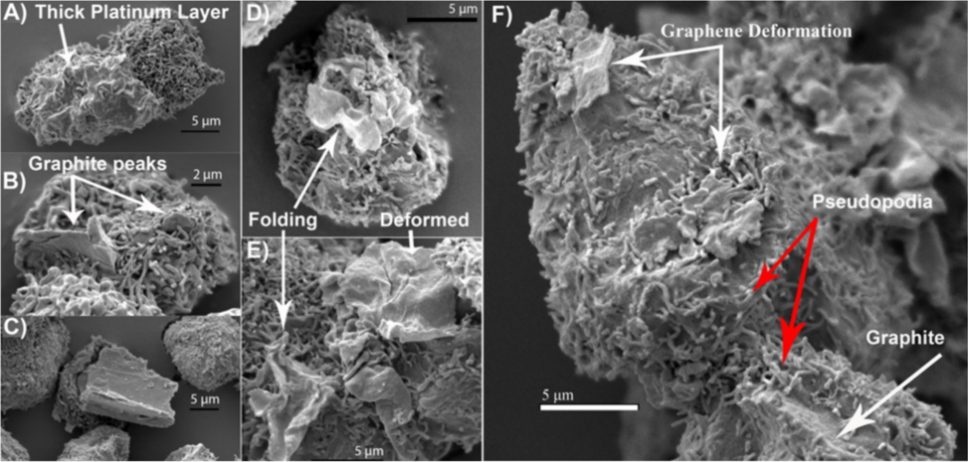
**SEM images of the interactions of completely exfoliated SGS and partially exfoliated SGS (i.e., graphite).** With the surface of SNU449 (**A**, **B**) and Hep3B (**C** to **F**) liver cancer cell lines.

Figure 4 depicts high-resolution SEM images of both SNU449 and Hep3B cancer cells after exposure to SGS at a concentration of 10 μg/ml for 24 h. Control samples (no SGSs) are shown in the Additional file 1: Figure S6. All samples were first coated with a 35-nm layer of platinum before imaging. The cells were approximately 10 to 25 μm in diameter and heterogeneous in nature. Figure 4A showed what is likely to be variability in surface coating of the platinum layer. When comparing the left and right images of the SNU449 cellular structures in Figure 4A, the left side has what looks like a thicker layer of platinum, which seems to be filling more of the space between adjacent pseudopodia structures. Comparing Figure 4A and Figure 4B, it can clearly be seen that a relatively large structure is protruding out of a SNU449 cell in two locations. These structures appear to be graphite (i.e., multiple stacked SGS) of thickness approximately 500 nm which the cell has internalized. Figure 4C depicts another large nanoplatelet of stacked SGS, which is effectively compressing a Hep3B cell and deforming the cellular structure. Figure 4D and Figure 4E are the most interesting figures since they display evidence of cellular internalization, folding, and compartmentalization of SGS.

In Figure 4D, it appears as if the Hep3B cell is actively internalizing multiple, stacked SGS of height approximately 35 nm, but is most likely a single SGS which looks thicker due to the platinum layer. The folding phenomenon is also evident in Figure 4E where folding of SGS can be seen in the bottom left corner and bottom midsection of the image, as indicated by the white arrows. There is also evidence of slightly deformed SGS on top of the cellular surface in the upper right-hand section. Finally, Figure 4F depicts the images of both SGS deformation and internalization of large pieces of graphitic materials. The appearance of pseudopodia over the surface of the SGS is indicated by the red arrows.

**Figure 5:**
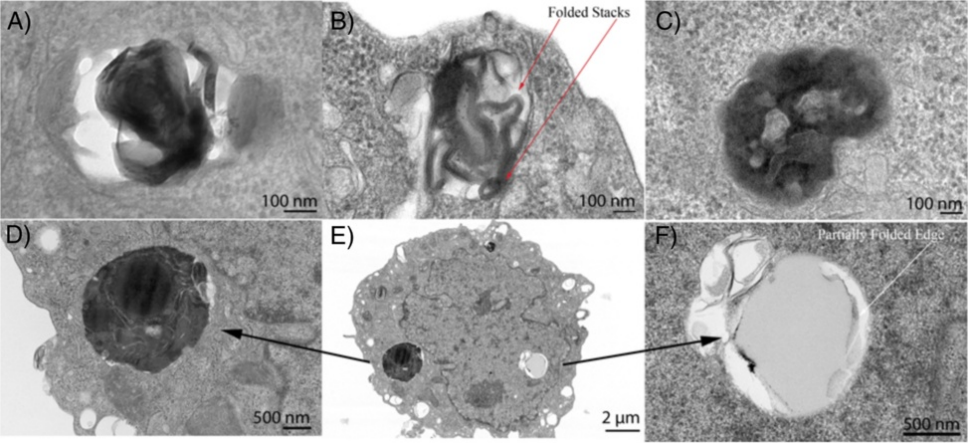
**SGS Internalization within Hep3B cancer cells.** TEM images of internalized carbonaceous material and SGSs within Hep3B liver cancer cells (**A** to **F**). Figure 4D,E,F is of the same cell

Cellular internalization of the SGS using microtome high-resolution TEM was then investigated, as shown in Figure 5. Uranyl acetate was used as a negative staining agent. Although single-sheet graphene should appear close to transparent in TEM imaging, we believe visualization of the SGS in the TEM images is due to uranyl ions binding to the functionalized graphene sheets (which would result in a darker image) or that they are stacked graphene layers which are reducing the optical transparency. From the outset, we suspected that there was some cellular internalization of submicron-sized amorphous carbonaceous materials present in the initial graphite material from which the SGS were obtained. Evidence of this can be found in the Additional file 1: Figure S1. Furthermore, Figure 5A,C indicates cellular internalization of these materials since there is no evidence of structural uniformity or stacking, which can usually be seen for graphene by TEM at this resolution [25]. However, Figure 5B clearly shows compartmentalization of SGS, and closer examination reveals a network of lines (red arrows) throughout this structure, which look exactly like the folded graphene sheets previously reported by A. K. Geim et al. [25]. A magnified view of this key figure is shown in Additional file 1: Figure S7.

Figure 5D,F shows close up images of two areas of Figure 5E to reveal a stained black circular particle (Figure 5E) and a more transparent, slightly smaller, circular particle (Figure 5F). As these particles are of the same diameter as the SGS previously characterized, they are likely SGS that have internalized into the cell without folding or compartmentalization. As previously indicated, the large difference in contrast between these two SGS structures could be due to uranyl ions binding to the functionalized SGS or due to multiple stacked graphene layers.

**Figure 6:**
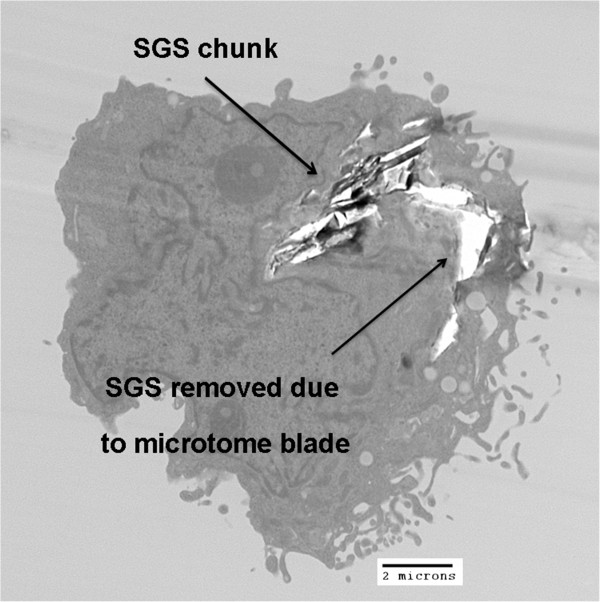
**TEM image of microtome cutting artifacts caused by SGS inside a SNU449 cell.** It is likely that some large chunks of graphite and/or SGS have been dislodged from the transparent region in the top right corner of the image.

It should be noted that the cellular internalization of large SGS caused artifacts in some instances during the microtome procedure. This can be seen in Figure 6 where there is a large area of internalized SGS adjacent to a completely transparent ‘hole’. This hole is most likely caused by the microtome blade contacting the SGS and removing the structure from the cellular cytoskeleton (thus leaving behind an SGS footprint). There is also some evidence of this in Figure 5A where the carbonaceous NP seems to have been dislodged from its initial position, leaving behind a transparent hole in the left image. This result also serves as good evidence of the cells’ ability to internalize relatively large pieces of graphite yet still remain healthy.

**Figure 7:**
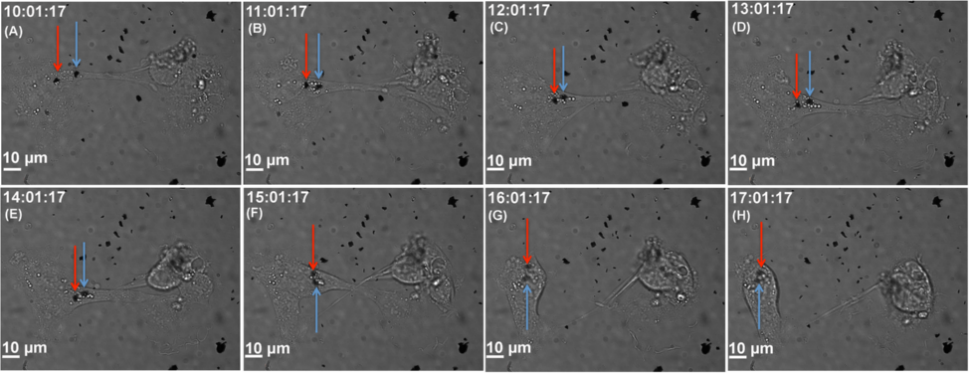
**Optical bright**-**field images of SGS internalization within Hep3B cancer cells across a 17-h period.** Two SGS particles of diameters approximately 2 μm, indicated by red and blue arrows, are shown to be internalizing approximately 10 h after exposure to SGS of concentration 10 μg/ml. The two cells visible seem to be undergoing cell division. (**A** to **H**) Time points at 10 to 17 h, in 1-h increments.

Using real-time bright-field optical microscopy, we could also track the internalization of SGSs in liver Hep3B cells as a function of time (over a 17-h period). As can be seen in Figure 7, when looking at snap shots from approximately 10 to 17 h, there were two large SGS (indicated by red and blue arrows) which became attached to the cell membrane and gradually internalized into the cell - as is evidenced by the loss of resolution and blurred nature of the SGS images. Furthermore, the cell retracted to undergo mitosis once the trapped particles are internalized. (Figure 7E,F,G,H, full movie also available in the Additional file 2: Hep3B SGS movie and Additional file 3: Hep3B control movie).

Given that graphene is thought to be the hardest material known [3], it is counterintuitive to believe that liver carcinoma cells are capable of folding and compartmentalizing graphene sheets. However, if these sheets contained structural defects such as point defects, single vacancies, multiple vacancies, carbon adatoms, dislocation-like defects, or edge defects, as extensively reviewed by Banhart et al. [26], the cells may be able to fold the sheets, one at a time, along these defect lines (in a ‘shedding nature’) and compartmentalize them within phagosomes or vesicles using reasonably low-energy processes. The defect content of the SGS, in relation to the starting graphite material, can be indicated by the relative intensity of the Raman D band to G band ratio, located at approximately 1,350 and 1,580 cm^−1^, respectively [27]. Although the synthesis procedure and Raman characterization shown in Additional file 1: Figure S2 shows a weak D band enhancement after exfoliation due to functionalization of the graphitic edges, it remains unclear as to what defects, if any, are inherent to the graphene nanoplatelets.

## Conclusions

We have investigated the cytotoxicity and internalization of highly exfoliated, water-soluble SGSs when exposed *in vitro* to highly aggressive human liver cancer cells (SNU449 and Hep3B). Both MTT and WST-1 colorimetric assays displayed a similar concentration- and time-dependent cytotoxicity profile for concentrations of 0.1 to 10 μg/ml. These trends were also evident from LDH observations. However, the SGSs seemed to be toxic to both cell lines at the highest concentration of 100 μg/ml. We have also observed an interesting cellular internalization phenomenon for graphene materials for the first time. The cancer cells were capable of internalizing relatively large SGSs with diameters comparable to the cells themselves as well as smaller SGS having heights indicative of single graphene sheets. Although not conclusive, there is evidence to suggest that due to graphene structural defects, the cancer cells are also able to actively fold and compartmentalize these sheets. We speculate that the findings reported here may encourage the development of SGSs for applications in drug delivery, medical imaging, and even hyperthermic cancer therapy by NIR and/or radio frequency heating. To date, such applications have been explored for more rigid carbon nanostructures such as fullerenes [28] and nanotubes [29-32], but a non-toxic, more flexible (foldable), and larger surface-area material as provided by graphene offers an alternative design strategy.

## Abbreviations

AFM: Atomic force microscopy; DMSO: Dimethyl sulfoxide; FACS: Fluorescence-activated cell sorting; GO: Graphene oxide; LDH: Lactate dehydrogenase; MTT: 3-(4,5-dimethylthiazol-2-yl)-2,5-diphenyltetrazolium bromide; NIR: Near-infrared; NPs: Nanoparticles; PI: Propidium iodide; PBS: Phosphate-buffered saline; PS: Phosphatidylserine, SGSs, sulphonated graphene sheets; TEM: Transmission electron microscopy; TGA: Thermogravimetric analysis; WST-1: WST-1[2-(4-iodophenyl)-3-(4-nitrophenyl)-5-(2,4-disulfophenyl)-2*H*-tetrazolium; XPS: X-ray photoelectron spectroscopy

## Competing interests

The authors declare that they have no competing interests.

## Authors’ contributions

SJC conceived the study, interpreted the results, guided the contributing authors in their research, performed the optical bright-field imaging (alongside MR), and wrote the manuscript. MR performed the MTT assay study, helped with the TEM/SEM imaging, and worked with SJC on the optical bright-field imaging studies. BTC carried out the LDH assay. OK synthesized and supplied the SGSs. KM and WDK performed FACS on the SNU449 cell line. MAC performed the AFM imaging of the SGSs. WEB, LJW, and SAC participated in the design of the experiments, acted as mentors for the authors, and extensively reviewed the manuscript. All authors read and approved the final manuscript.

## Supplementary Material

Additional file 1 **Supplementary information.** Figure S1: AFM images of SGSs, Figure S2: Raman spectra, Figure S3: XPS spectra, Figure S4: TGA of completely exfoliated SGSs, Figure S5: FACS analysis, Figure S6: SEM image, and Figure S7: magnified view of Figure 5B (maintext). Click here for file

Additional file 2 **Hep3B SGS movie.** Movie sequence of SGS internalization over a 17-h time period. Cell lines are Hep3B. Click here for file

Additional file 3 **Hep3B control movie.** Movie sequence of Hep3B control (no SGS exposure) across a 17-h time period. Click here for file
