# Supplementary material for: Cytotoxicity and variant cellular internalization behavior of water-soluble sulfonated nanographene sheets in liver cancer cells
Source: Nanoscale Res Lett. 2013 May 2;8(1):208. doi: 10.1186/1556-276X-8-208 (PMC3663679; doi:10.1186/1556-276X-8-208)
Supplement: Additional file 1 — Supplementary information. Figure S1: AFM images of SGSs, Figure S2: Raman spectra, Figure S3: XPS spectra, Figure S4: TGA of completely exfoliated SGSs, Figure S5: FACS analysis, Figure S6: SEM image, and Figure S7: magnified view of Figure 5B (maintext). [file 1556-276X-8-208-S1.pdf]

Supporting Information

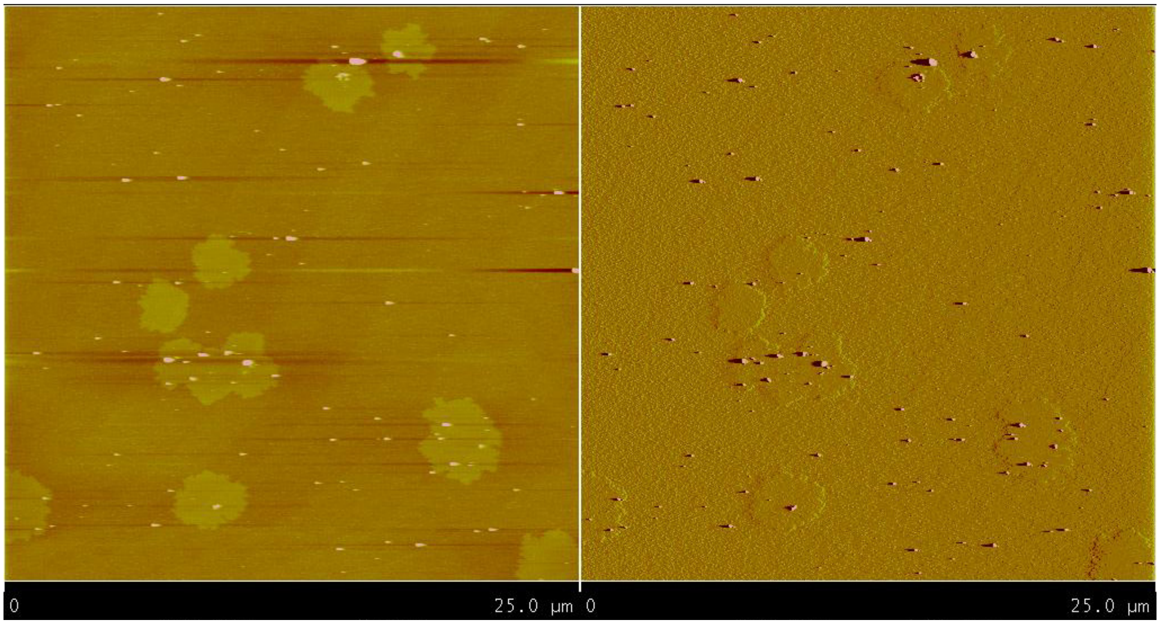

Figure S1: AFM images of SGSs. Left image depicts completely-exfoliated SGSs of diameter 3 μm to 5 μm and height ~1 nm. Right image is identical to left image but expresses the Z-height in voltage. There is also evidence of amorphous carbon material, which can be seen as small white spots.

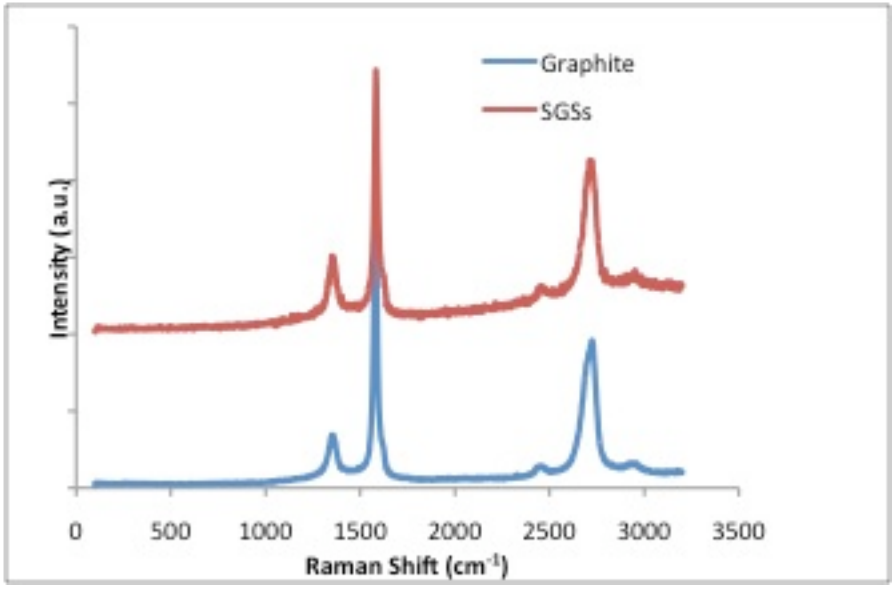

Figure S2: Raman spectra of initial graphite material (blue) and completely-exfoliated SGSs (red).

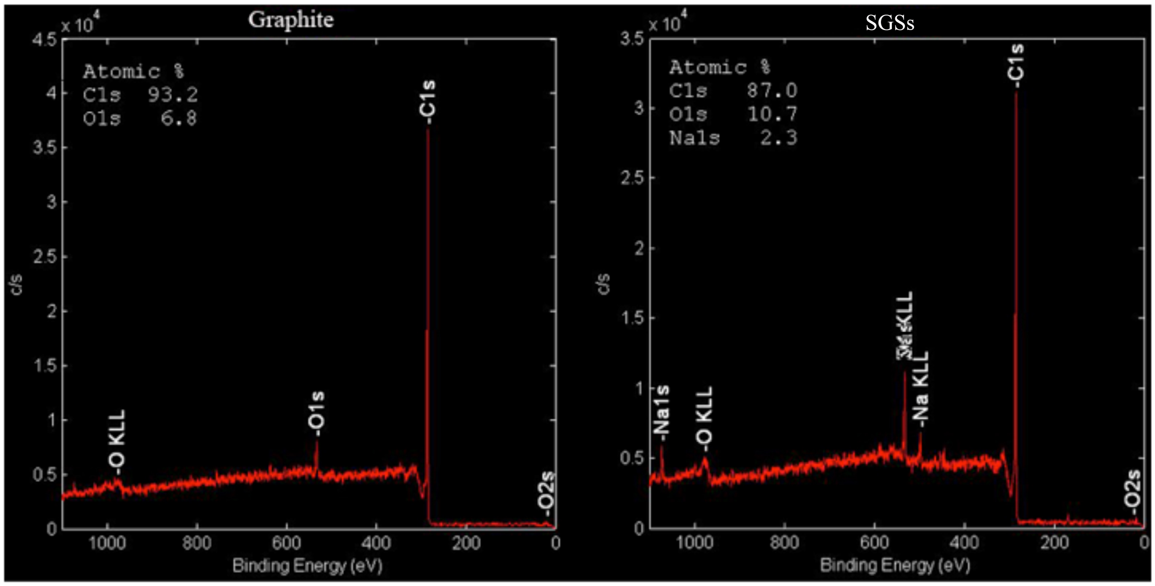

Figure S3: XPS spectra of the initial graphite material (left) and completely-exfoliated SGSs (right). Right spectra shows the presence of the sodium 1s peak at ~1070 eV, as well as a small sulphur 2p peak at ~168 eV.

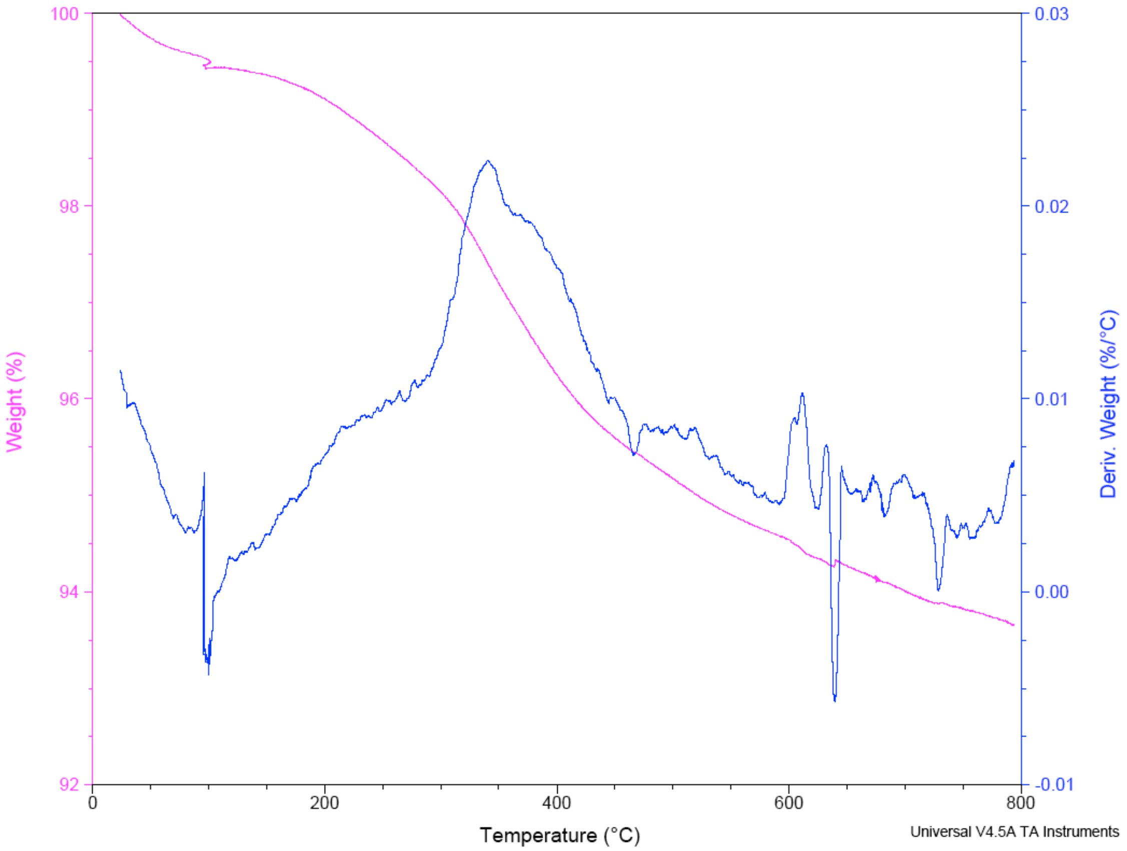

Figure S4: TGA of completely-exfoliated SGSs indicating a weight loss of 6 % over the temperature range 0 ºC to 800 ºC.

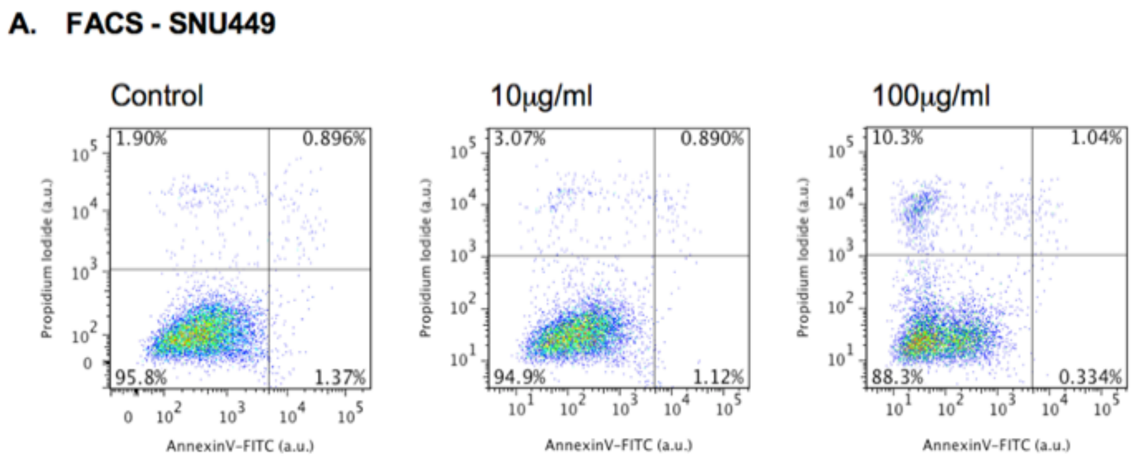

Figure S5: FACS analysis (50,000 events) of the SNU449 cancer cell line at various SGS concentrations after 24 hours. The majority of cells are viable (~93 ± 2.4 %) but depict a slight increase in the intensity of positive-PI-stained cells suggesting minimal cell membrane damage. No strong apoptosis-mediated pathways are observed.

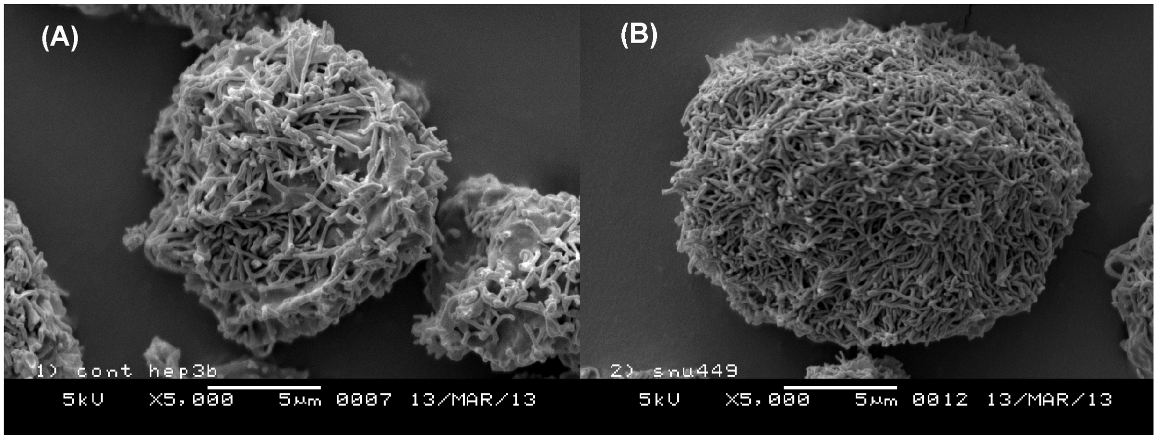

Figure S6: SEM image of control (no SGS) Hep3B and SNU449 cell lines after 24 hours (A and B, respectively).

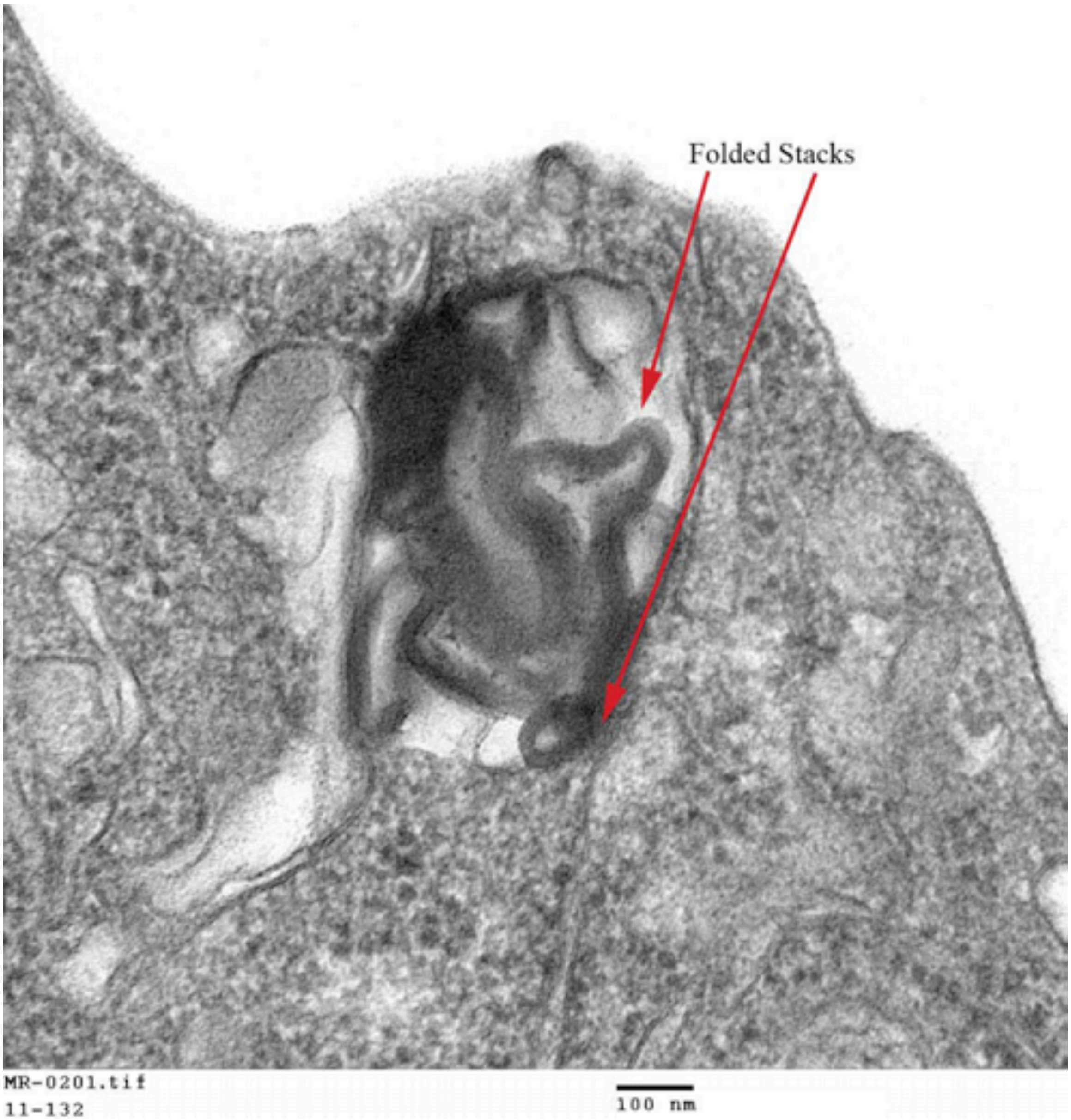

Figure S7: Magnified view of Figure 5b (main text) showing a cellular vesicle containing folded SGSs.

Movie S8: Real-time bright field optical imaging video of Hep3B cells treated with 10 μg/ml SGSs over a period of ~17 hrs.

Movie S9: Real-time bright field optical imaging video of control Hep3B cells over a period of ~17 hrs.
